# Barriers to preventive care utilization among Hong Kong community‐dwelling older people and their views on using financial incentives to improve preventive care utilization

**DOI:** 10.1111/hex.13256

**Published:** 2021-05-05

**Authors:** Qiuyan Liao, Wingyan Lau, Sarah McGhee, Maurice Yap, Rita Sum, Jun Liang, Jinxiao Lian

**Affiliations:** ^1^ School of Public Health The University of Hong Kong Hong Kong Hong Kong; ^2^ School of Optometry The Hong Kong Polytechnic University Kowloon Hong Kong; ^3^ Department of Family Medicine and Primary Health Care New Territories West Cluster, Hospital Authority Hong Kong

**Keywords:** barrier, financial incentive, older people, preventive care, qualitative study

## Abstract

**Background:**

Financial incentive is increasingly used as a mean to promote preventive care utilization (PCU), but the current Elderly Health Care Voucher Scheme (EHCVS) in Hong Kong is ineffective for encouraging PCU.

**Objective:**

To explore the older people's barriers to PCU and their views on financial incentive, including EHCVS, for improving private PCU.

**Design and setting:**

Focus‐group discussions were conducted in community elderly centres located in five districts of Hong Kong.

**Participants:**

Community‐dwelling older people aged 60 years or above.

**Results:**

Lack of understanding about preventive care and low awareness of the need for preventive care were key factors for the low motivation for PCU. Uncertainty over the level of service fee charged and concerns over service quality hindered the choice of using the private service providers under the current EHCVS. Financial incentives specific for preventive care services were thought to be cues to actions and guides for service promotion. However, some flexibility in service coverage and a set time limit of the financial incentives were preferred to accommodate individual needs.

**Conclusions:**

Apart from promoting knowledge of preventive care, official monitoring for service fee and quality is important for empowering older people to choose private service providers for preventive care. Financial incentives for preventive care services should be more specific to cue service promotion and uptake of preventive care while maintaining flexibility to accommodate individual needs.

**Patient or public contribution:**

Participants were recruited using purposive sampling with the coordination of community elderly centres. Data were analysed using thematic coding.

## 1 INTRODUCTION

The global population is ageing rapidly with increases in life expectancy and declining fertility.^1^ As in many jurisdictions, the Hong Kong (HK) population aged 65 years or older is projected to increase from 17% in 2015 to 33% in 2064.^2^ Population ageing is associated with increasing health care demand, in particular among older people with non‐communicable chronic diseases (NCDs), putting pressure on the health‐care system and increasing family caregiver burden.^3^ Appropriate preventive care utilization (PCU) could enhance early detection and treatment of disease complications and therefore improve quality of life and reduce long‐term health​ care costs.^4^, ^5^ However, PCU rates among older people remain low in HK. Older adults are recommended to have regular health checks to identify risk factors or complications in their early stages, enhance active ageing and reduce future costs to the health‐care system.^6^ However, only about 40% of older people in HK have regular check‐ups.^7^ A similar uptake was found in the National Health Service (NHS) Health Check in the United Kingdom, much lower than the original aspiration of 75% uptake rate.^8^, ^9^ It was estimated through modelling that the NHS Health Check Programme could prevent 300 premature deaths and 1000 cases of cardiovascular disease, dementia and lung cancer each year in England.^10^

HK has a mixed medical economy with inpatient services largely provided by public hospitals (90%) and primary care largely from the private sector (70%), paid out‐of‐pocket.^11^ Public primary care charges around HK$50 (~US$6/ ~£5) per public general outpatient visit,^12^ while a visit to one of more than 3700 Western medical practitioners’ clinics can cost five times as much.^13^, ^14^ Other health‐care professionals such as Chinese medicine practitioners, dentists, nurses, physiotherapists, occupational therapists and optometrists also provide private services, and while private services are available to anyone, users bear the full cost since most are not subsidized by government.^14^ In 2009, the HK Government launched an Elderly Health Care Voucher Scheme (EHCVS) to encourage older people to purchase private preventive care and chronic disease management services and to reduce the burden on the public sector.^15^, ^16^ The voucher is worth HK$2,000 (~US$258 / ~£202) annually, can be used to pay for services offered by enrolled practitioners, and unspent vouchers can be accumulated up to HK$8,000 (~US$1,032/ ~£809).^15^ However, a recent study found that the voucher scheme did encourage use of private services for acute illness but not for preventive care or chronic disease management.^17^ We therefore sought to understand how financial incentives might encourage PCU by obtaining information from the users’ perspectives. With a current lack of any information from that perspective, we designed a qualitative study to explore factors contributing to the underuse, by older people, of private preventive care services and their opinions on financial incentives. The specific research questions were as follows:

1. What are some of the barriers for older people in using private preventive care services?
2. What are older people's views on financial incentives for PCU through private care providers?

The results from this study can provide useful information from users’ perspectives for developing financial incentives to encourage PCU in older people.

## 2 METHODS

### 2.1 Participants and subject recruitment

Between May and June 2019, five focus‐group discussions (FGDs) were conducted in elderly centres located in five out of 18 administrative districts in HK, namely Kwai Tsing, Northern, Sha Tin, Tseung Kwan O and Yau Tsim Mong, with all having sufficient availability of private primary care providers within the district.^18^ These were the first five centres who agreed to participate when we sent out the invitation to all 18 districts and were the only centres included since we used data saturation (see later) as our guide to sample size.

Community‐dwelling people aged 60 years or older were invited, by the centre staff of the selected elderly centres, to participate in the study. The centre provided the list of potential participants to the project principal investigator who selected six to ten people from each centre for the FGD, using purposive sampling to ensure a spread of sex, age, educational attainment and family income across the five FGD. Exclusion criteria were not being a Hong Kong–resident, unable to give consent to participate in the study or unable to communicate adequately due to linguistic or cognitive difficulties. Each FGD was held in a quiet room in the centre.

### 2.2 Topic guide and data collection

After giving written consent, participants completed a brief questionnaire about their demographics and were assured about anonymity and confidentiality of data. The topic guide was modified after the first FGD. This modified version (Appendix 1) was used for the four following discussions. The interview comprised three parts. First, the discussion began with questions about what preventive care is and participants’ patterns of use. Reasons for not using preventive care were queried by the moderator. The second part explored how participants used their current EHCVS and whether they might use it for preventive care. The third part explored participants’ views about what financial incentives might encourage PCU. All discussions lasted around 60 minutes, were moderated by two researchers and audio‐taped with a research assistant taking notes. Each participant received HK$100 (~US$13) shopping voucher as a travel allowance.

The number of FGD conducted was determined by data saturation, defined as no new data emerging from the last two FGD.^19^ In our study, we identified no new data at the fourth FGD and confirmed data saturation by conducting the fifth FGD.

The study received ethical approval from the Human Subjects Ethics Sub‐Committee (HSESC) of the Hong Kong Polytechnic University (HSESC Reference Number: HSEARS20180629002).

### 2.3 Data analysis

All interviews were transcribed verbatim for data analysis. Accuracy of transcription was checked before data analysis by re‐listening to the recordings. Data were analysed using thematic coding.^20^ A thematic coding scheme was first developed by two experienced researchers by reading through the five transcripts, and then, it was refined by discussion with the research team. One experienced researcher and one research assistant both coded all the data, independently, following the coding scheme but allowing new codes to emerge from the data. Codes with Cohen's kappa coefficient of lower than 0.6 indicate low inter‐rater reliability^21^ and were solved by joint discussions among three researchers to reach a consent in coding. During the coding process, constant comparison methods were followed to compare the similarities and differences of existing codes and newly emerged codes.^22^ Theoretical categories were developed by clustering similar codes, and related categories were linked to develop themes relevant to the research questions. QRS NVivo 12.0 was used for data analysis.

## 3 RESULTS

There were 37 participants across the five focus groups with a group size of 6 to 10. More participants were female, had a monthly family income of less than HK$10,000 (~US$1,282) and had at least one NCD; age and educational attainment were more heterogeneous (Table 1). Relevant themes are presented below, categorized by the two research questions:

**TABLE 1:** Characteristics of participants (N = 37)

Characteristics	N	%
Sex		
Male	8	21.6
Female	29	78.4
Age group		
60‐64	3	8.1
65‐69	7	18.9
70‐74	11	29.7
75‐79	5	13.5
80‐84	8	21.6
85+	3	8.1
Education level		
No schooling/Pre‐primary	5	13.5
Primary	17	45.9
Secondary lower (F.1‐3)	7	18.9
Secondary upper (F.4‐5)	3	8.1
Sixth form	2	5.4
Post‐secondary	2	5.4
Degree or above	1	2.7
Monthly family income (in Hong Kong dollars)		
Less than $2,000	3	8.1
$2,000‐$3,999	20	54.1
$4,000‐$5,999	3	8.1
$6,000‐$7,999	4	10.8
$8,000‐$9,999	0	0.0
$10,000‐$14,999	2	5.4
$15,000‐$19,999	0	0.0
$20,000‐$24,999	1	2.7
Refuse to answer/ Don't know	4	10.8
Hypertension		
No	12	32.4
Yes	25	67.6
Don't know	0	0.0
High cholesterol		
No	19	51.4
Yes	16	43.2
Don't know	2	5.4
Diabetes		
No	28	75.7
Yes	9	24.3
Don't know	0	0.0
Coronary heart disease		
No	29	78.4
Yes	6	16.2
Don't know	2	5.4

### 3.1 Barriers to using preventive care services provided by private primary care practitioners

**FIGURE 1:**
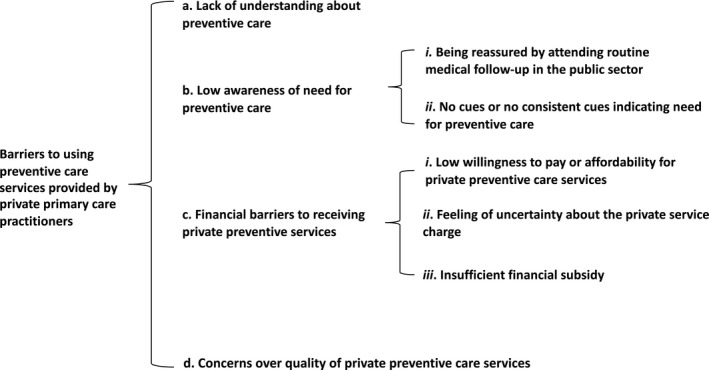
Research themes relating to barriers to using preventive care services provided by private primary care practitioners among older people

Most participants reported infrequent use of preventive care services. When the moderator asked the question, ‘do you have regular health check‐ups in private clinics or hospitals?’ only one interviewee responded that they had regular health check‐ups, across the five FGD. Four themes emerged for what contributed to underuse of preventive care services from the private sector (Figure 1).

#### 3.1.1 Lack of understanding about preventive care

Most participants stated having little knowledge about preventive care. Some seemed unable to distinguish preventive care from curative care and reported that they would only use ‘preventive care’ services when they perceived a ‘need’ of their body. Some reported a need for more information about preventive care.

Moderators: *‘Do you know what preventive care is?’*

Interviewee 1: ‘*I do* *n't* *know …’* (Focus Group 2, aged 70, female)

Interviewee 2: *‘Preventive care… I don'tknow…’* (Focus Group 3, aged 63, female)

Interviewee 3: ‘*Could the government…organize (some talks) or activities…to introduce what prevention and treatment is? I have to say that this is the first time I have heard of such term (laugh…)’*. (Focus Group 3, aged 78, male)

#### 3.1.2 Low awareness of need for preventive care

A few participants were able to explain the general concept of preventive care or name some specific preventive care services, but were not clear about what preventive care services they would need. Some participants perceived no need for preventive care services and put curative care at a relatively higher priority. Two major categories explained this theme:

##### Being reassured by attending routine medical follow‐up in the public sector

Participants who had routine follow‐up of chronic conditions in the public sector considered this a way to receive ‘comprehensive’ preventive care and therefore perceived no need for preventive services from the private sector. They seemed reassured by the check‐ups in routine consultations and believed the doctors could decide what further check‐up they needed.

Interviewee: ‘*For preventive care, I usually get this done at the nearby public hospital, where services are fully covered by the hospital. They are responsible for measuring our blood pressure, prescription, eye examination, blood test…and many more, simply all of them’. (Focus Group 4, aged 70, female)*

##### No cues or no consistent cues indicating need for preventive care

This seemed to be a major reason why preventive care was deemed a low priority. It also reflects a general lack of understanding about the purpose of preventive care. Perceiving no physical cues (eg pain) and, in particular, no consistent physical cues was misinterpreted as ‘no need’ for preventive care services.

Moderator: *‘So you believed it is not necessary (to get a physical examination)?’*

Interviewee: *‘yes, I don'thave abnormal feeling in my body. That'swhy I did not go for it (physical examination)’*. (Focus Group 5, aged 77, female)

#### 3.1.3 Financial barriers to receiving private preventive care services

When discussing seeking preventive care services, the private providers were not a preferred choice for most participants due to financial concerns. Three financial barriers to seeking private preventive care services were identified:

##### Low willingness to pay or affordability for private preventive care services

Participants generally indicated a low willingness to pay for preventive care services provided by the private sector because they had got used to receiving health‐care services from the public sector with a relatively low out‐of‐pocket expense. Participants of lower socio‐economic status indicated difficulty in affording preventive care services from the private sector.

Interviewee: ‘*To be honest, people of lower social class rarely get a dental scaling. I* *t's* *likely that the middle class would use more of this service more. To be honest, for our age group, we…are from a lower social class and rarely go for dental scaling. Scaling means removing tartar, right? …* *we* *just did* *n't* *really go for this… But we will go if our tooth aches!’* (Focus Group 2, aged 67, male)

##### Feeling of uncertainty about the private service charge

Almost all participants indicated feelings of uncertainty regarding the private service charge. Most expressed discontent about the higher service fees charged by the private sector when health‐care vouchers were used and the insufficient transparency of their charges. This also promoted a sense of need for more government monitoring and regulation.

Interviewee: ‘*I realize that the private clinics tend to state a different selling price for those who use the health care voucher, rather than paying by cash. They usually charge more for those who use healthcare vouchers based on my observation’*. (Focus Group 1, aged 81, female)

##### Insufficient financial subsidy

Although health‐care vouchers were provided with a goal of encouraging more PCU, most participants indicated not being able to use this financial subsidy for preventive care as the vouchers were already insufficient for their curative needs. Even with unspent vouchers, participants tended to save up the vouchers for curative care rather than spending the vouchers on private preventive care services. This habit seems to be related to a perception that ageing is linked to poor health status, and therefore, money should be saved for ‘urgent’ health problems.

Interviewee 1: ‘*A serious disease can use up all your vouchers… Once you got a major disease, then you would be hospitalized, and thereafter you have to see a doctor or use other services. When you feel very sick, why would you wait for seeing a doctor (in a public hospital)?’* (Focus Group 4, aged 64, female)

Interviewee 2: ‘*Agree! You simply just do* *n't* *have enough money. It (the voucher amount) has already been insufficient to cover the expenditure of medical treatment. Why would you use it (the health care voucher) for a check‐up?’* (Focus Group 3, 70‐74, male)

#### 3.1.4 Concerns over quality of private preventive care services

Some participants indicated concerns over the quality of preventive care services provided by the private sector.

Interviewee: ‘*I distrust the quality of the private clinics when it comes to eye examination…I did not trust them. Therefore, I went to the Prince of Wales Hospital (a public hospital) for eye examination. I have received eye examination in the Prince of Wales Hospital and I feel more relieved after checking my eyes there’*. (Focus Group 1, aged 88, female)

### 3.2 Views on financial incentives for preventive care

**FIGURE 2:**
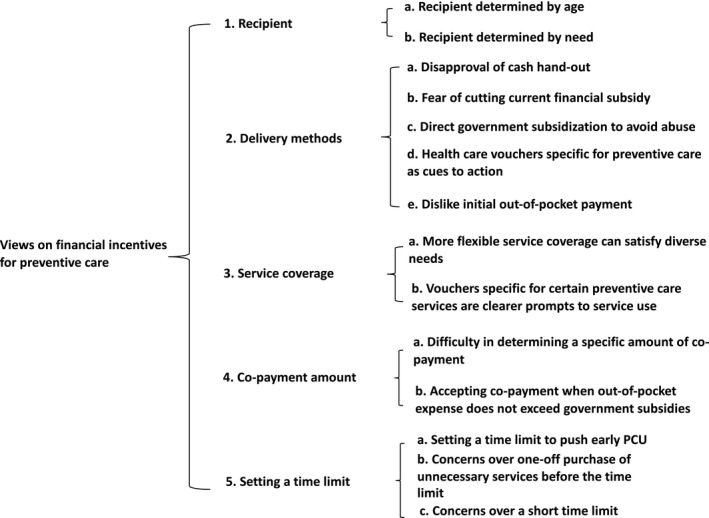
Research themes relating to views on financial incentives for preventive care

Participants were encouraged to discuss their views on important characteristics of financial incentives derived from the literature^23^, ^24^, ^25^, ^26^, ^27^ and in what ways these attributes could influence their decision for PCU. The research themes and categories are organized and presented based on characteristics of financial incentives for PCU that were specifically explored during the interview (Figure 2).

#### 3.2.1 Recipient

Two main themes emerged when participants were asked about who should be eligible for financial incentives for preventive care services.

##### Recipient determined by age

There was a general consensus that age was important to determine eligibility for receiving financial incentives for preventive care. Various reasons were given when discussing why age should be an entry criterion due to different understanding about the purposes of preventive care. Most participants believed that older people needed the financial subsidies more than the young people due to a perception of ageing being associated with poorer health status. Several participants, who seemed to have a better understanding of preventive care, believed that the entry age should be younger for early uptake of the preventive measures. Some participants suggested promoting PCU around age 60, five years younger than the current eligible age criterion of 65 for EHCVS.

Interviewee 1: ‘*I* *t's* *a must for us in the 60s… We may have this or that problems. All these problems need (health care vouchers to support seeking healthcare)’*. (Focus Group 2, aged 77, male)

Interviewee 2: ‘*Preventive measure should be done at a younger age (Cross talk, confusion). There is no point for prevention after we have experienced all these illnesses’*. (Focus Group 3, aged 70‐74, male)

##### Recipient determined by need

Eight participants believed that the financial incentive for preventive care should be provided based on need. Financial status and health status were believed to be the two main indicators for need. These participants also mentioned age as an indicator for need mainly because ageing was perceived to be linked to poor health status and lower financial capacity. One participant mentioned that patients who had regular medical follow‐up in public hospitals should not be eligible for financial incentives for preventive care because the regular follow‐up was believed to provide preventive care.

Interviewee 1: *‘People who don'thave enough money would need this (financial incentive), right?’* (Focus Group 2, aged 73, female)

Interviewee 2: ‘*Exactly, those who have a lot of money would* *n't* *need to be sponsored by the government’*. (Focus Group 2, aged 76, female)

Interviewee 3: *‘I have wanted to mention one point for a while. The health care voucher…The health care voucher is basically useless for those chronic patients who regularly visit Northern hospital (a public hospital) (for follow‐up appointment). They then accumulate the voucher amount year by year. If they don'tuse it, the government will not provide more (subsidy) to them. Then, isn'tthis a waste of the voucher?*.*.’* (Focus Group 3, aged 66, female)

#### 3.2.2 Delivery methods

Different ways of delivering financial incentives for preventive care were discussed during the interviews including designating a proportion of the current health‐care vouchers for preventive care, increasing the amount of current health‐care vouchers, paying cash, and designing new health‐care vouchers specifically for preventive care and direct, full/partial government subsidies for preventive care services. Four main themes emerged relating to delivery methods.

##### Disapproval of cash handout

Almost all participants agreed that a cash handout was not effective in encouraging PCU due to the difficulty in monitoring how the cash was spent, and therefore, it would lead to abuse of the financial resources.

Interviewee: ‘*I think cash hand‐out is useless because you (the government) cannot monitor how I use them. For example, people could use it to buy dried fish maw when it is cheap’*. (Focus Group 1, aged 74, male)

##### Fear of cutting current financial subsidy

There was also a common opposition to allocating a proportion of the current health‐care voucher amount for preventive care. This was especially the case among those who perceived the current health‐care vouchers were insufficient for curative care.

Interviewee: ‘*Well, let me put it in this way. I would absolutely be in favor of this if I am free of disease. However, those who have diseases will be surely against such an idea. I mean, it is already not enough for seeing a doctor for treatment, and you said to allocate a part of the vouchers for prevention. What to prevent when he is already ill? This wo* *n't* *work’*. (Focus Group 2, aged 78, male)

##### Direct government subsidization to avoid abuse

Some participants preferred preventive services to be directly subsidized by the government because they believed that this could ensure that PCU was based on need. Some mentioned the examples of influenza vaccination and colorectal cancer screening, which were directly subsidized by the government as a good way to promote PCU.

Interviewee 1: ‘*The colorectal cancer screening programme (direct subsidized by the government) is implementing pretty well to prevent the disease’*. (Focus Group 3, aged 66, female)

Interviewee 2: *‘I’m in my 50s and have received the (colorectal cancer) screening’*. (Focus Group 3, aged 70‐74, male)

Interviewee 3: ‘*I think the government has done a great job with providing this service [directly subsidized vaccination]’*. (Focus Group 4, aged 64, female)

##### Health care vouchers specific for preventive care as cues to action

Participants generally believed that health care vouchers designed specifically for preventive care can be more effective to encourage PCU. One important reason was that the more specific vouchers could work as a cue to prompt action.

Interviewee: *‘I don'treally care about the amount; I would love to use the voucher if I were given one! At least you could use it for preventive care. For example, it can be used for preventive check‐up. If the voucher is designated for a specific service, like dental check‐up, I would definitely use it for dental check‐up’*. (Focus Group 2, aged 76, female)

##### Dislike initial out‐of‐pocket payment

When discussing delivery methods, participants also raised the issue of delivery time. There seemed to be a consensus that the financial subsidies should be provided before or at the time of service use. Initial out‐of‐pocket payment by the user was generally not favoured because of perceived financial difficulties to pay for the services upfront.

Interviewee: *‘If the government would subsidize one certain health service, I would prefer receiving the subsidy at the point of service registration rather than later. I mean, what if I don'thave sufficient money to pay for the service? When I register for the service, the government can provide the subsidies to me, and I don'thave to wait till they repay me after I have utilized the service. It can be problematic if I didn'thave sufficient money to pay…’* (Focus Group 2, aged 70, female)

#### 3.2.3 Service coverage

Participants also discussed whether the financial incentives should be specific for particular preventive services (eg vouchers for physical examination) or for any preventive care services. Participants traded off the flexibility of service coverage of financial incentives, which generated two main themes:

##### More flexible service coverage can satisfy diverse needs

Some participants favoured more flexible service coverage because they could choose the services based on their need.

Interviewee: *‘It is better to be more flexible (not restrict the voucher for a particular preventive care services) … It'seasier for us because you never know what specific type of services you would need’*. (Focus Group 5, aged 72, female)

##### Vouchers specific for certain preventive care services are clearer prompts to service use

Some participants, however, believed that having a designated voucher would be more effective to prompt actions because the voucher specific for certain preventive care helps clarify needs.

Interviewee: *‘If you are given the voucher for preventing eye problems, you should definitely use it for eye preventive care. If the voucher is for preventing a dental problem, then it should be spent on dental appointment. We should use the resource provided to us for prevention’*. (Focus Group 2, aged 76, female)

#### 3.2.4 Co‐payment amount

Co‐payment amounts for preventive care services were discussed during the interview, comprising two themes:

##### Difficulty in determining a specific amount of co‐payment

Participants generally had difficulties in specifying an acceptable amount of co‐payment for preventive care services due to uncertainty about the service fees charged by the private sector and different affordability of older people.

Interviewee: *‘This (how much we can share to pay for the service) is very difficult to say, since the (private)doctorscharge a lot’*. (Focus Group 3, aged 67, female)

##### Accepting co‐payment when out‐of‐pocket expense does not exceed government subsidies

A proportionate co‐payment method was suggested by several participants. Although participants accepted different proportions of out‐of‐pocket expense for various preventive services, it appeared that participants were more accepting of a co‐payment if the government paid at least 50% of the total service fee.

Interviewee: *‘For example, if it (the physical examination) costs HK$5000 and the government can subsidize for HK$3000‐4000, then I would just need to pay around HK$1000 or more. This would be acceptable’*. (Focus Group 4, aged 70, female)

#### 3.2.5 Setting a time limit

Participants were also encouraged to discuss whether a time limit should be set for using the financial incentive for preventive care. This generated three main themes:

##### Setting a time limit to push early PCU

Participants who agreed with setting a time limit believed that it can ‘push’ earlier PCU. One participant mentioned that missing the time to use the financial incentive could induce a feeling of regret.

Interviewee: *‘Actually, if you say, everyone should use it (the voucher) within one year and each year they can use it once, they would feel that they have missed out the opportunities if they don'tuse it’*. (Focus Group 3, aged 63, male)

##### Concerns over one‐off purchase of unnecessary services before the time limit

Participants who disagreed with setting time limits were concerned that it could encourage unnecessary use of the service in order to use the financial incentive.

Interviewee 1: ‘*This (setting time limit) would force people to buy things (before time limit)’*. (Focus Group 1, aged 65‐69, female)

Interviewee 2: ‘*Like what are reported in news, the majority would use it to purchase glasses (if there is time limit for using the vouchers)’* (Focus Group 1, aged 73, male)

##### Concerns over a short time limit

Several participants, though agreeing with time limits, had concerns over short time limits, and one suggested a limit of two years. The concerns over short limits included insufficient time to consider what services were needed or to accumulate funds for more costly services.

During the interviews, participants were also asked their opinions on whether a subsidized ceiling for the cost of specific services and an accumulation ceiling for the financial incentives should be set. Participants generally supported setting subsidized ceilings for specific services to manage the charges in the private sector but disagreed with accumulation ceilings.

Interviewee 1: *‘It is better to allow us to accumulate the voucher’*. (Focus Group 5, aged 81, female)

Moderator: *‘OK Do you think it needs to set a time limit for how long the voucher can be accumulated or it is better not to set such time limit?’* (Focus Group 5, Moderator)

Interviewee 2: ‘*It is better to allow us to accumulate for a longer period. Sometimes if you want to go for a dental check, the dental services are very expensive. The vouchers can be helpful’*. (Focus Group 5, aged 90, female)

## 4 DISCUSSION

This study identified barriers to PCU that are consistent with Anderson and Newman's framework of health‐care service utilization,^28^ which classifies factors that influence health‐care service utilization into predisposing factors, enabling factors and illness levels. For PCU among older people, lacking understanding about and perceiving no need for preventive care may predispose a low motivation for PCU. When seeking service providers, cost uncertainty and concerns over service quality (lack of enabling factors) impede choosing private health service providers. If people perceive good‐quality treatment at public hospitals, they will not be encouraged to use private care, particularly with the possibility of additional out‐of‐pocket expenditure.^29^, ^30^ There is also no immediate gratification (eg relieving symptoms) associated with PCU use (lack of illness‐related factors).

Adding to existing literature, our study identified contributors to perceiving no need for preventive care and financial barriers. Participants may determine need for preventive care based on somatic symptoms. This reflects a common misunderstanding about the goals of preventive care. In addition, since older HK residents commonly have routine chronic disease follow‐up in the public sector,^31^ this was perceived to be a way to obtain preventive care services, and therefore, there was no need for additional preventive care, especially when a visit to a private practitioner would cost around five times the cost of a visit to a public clinic.^12^, ^13^ There was a widespread feeling of uncertainty about charges in the private sector, in particular, perception of a tendency to charge more when vouchers were used. This led to a request for better governance of the voucher system. Compared to curative care, preventive care was a lower priority for using health‐care vouchers^17^ with no immediate gratification. Negative perceptions of ageing^32^ appeared to influence behaviour, and some would save unspent vouchers towards future curative care. In addition, some participants expressed concern over service quality of the private sector, which contributed to a low willingness to pay for private preventive services.^33^ Measures to increase trust in the service quality of the private sector are important in reducing barriers to PCU. With 70% primary care services provided in the private sector,^11^ private providers are widely distributed across HK providing easy access.^18^

We identified a common pattern of views on financial incentives for PCU; participants traded off advantages and disadvantages of flexibility in service coverage, delivery methods and time limits. The more flexible incentives, such as cash, were generally disapproved of. Concerns included difficulty monitoring how the incentives were spent and possible abuse. Less flexible incentives, such as vouchers designated for preventive care or with time limits and direct government subsidies, were preferred. Perceived advantages of less flexible, designated, vouchers were promoting preventive care, clarifying services available, encouraging early PCU and minimizing abuse, but concerns included short time limits for spending. This suggests that while people wanted a clearer guide to needed services and cues to push PCU, they also preferred to maintain autonomy in spending the incentives, based on self‐perceptions of need and financial status. Concerns over allocating a proportion of the current voucher amount to preventive care reflect that people did not want to lose autonomy in deciding how vouchers were spent—the phenomenon of loss aversion. To increase the acceptability of a co‐payment, the amount of incentive might be service‐specific and subsidy set to at least 50% of the total service fee, this awaits further testing in future studies.

Our study has several limitations. First, while we tried to ensure heterogeneity of participants’ characteristics, we encountered difficulties in recruiting males and those with higher socio‐economic status. Second, group discussions always risk omitting the views of quieter individuals but every effort was made to encourage everyone to voice their views. Third, this study only explored views on design of financial incentives from the perspective of older people (the demand side). Future studies should also consider the perspective of service providers (the supply side).

Our study flags several shortcomings, and possible solutions, in the alignment of policy intention with programme implementation. First, an education programme should be designed to promote understanding about preventive care, in particular, its goals. However, promoting knowledge of preventive care may not ensure a perception of need. Immediate benefits such as positive feedback after regular preventive check‐ups could provide immediate gratification of taking up preventive care. In the absence of internal cues (eg somatic symptoms) to prompt the need for preventive care, external cues such as electronic reminders (eg vaccination reminders) could be considered to alert potential users. While the public health‐care providers in HK play an important role in monitoring chronic illnesses, there are gaps in the preventive care services they offer. Since they often establish a trusting relationship with patients, the regular follow‐up consultation provides opportunity for encouragement to use private preventive care services. Private health‐care providers need to establish trusting relationships with older people. Charges for services should be transparent to reduce concerns about affordability. There might also be government‐sanctioned oversight of quality and fees.

More specific financial incentives for preventive care could be considered to prompt service‐seeking while some flexibility in terms of service coverage and time limit should be maintained to allow users room to decide how and when to use the subsidy. The amount of the incentive might be service‐specific, covering at least 50% of the total fee. The financial incentives should be provided before or at the time of service to avoid cash flow barriers. Finally, more positive perceptions of ageing could be promoted, consistent with wellness supported by regular PCU.^32^

## 5 CONCLUSION

Lack of understanding of preventive care and its importance, financial barriers and concerns over service quality all hinder use of the private sector for PCU in HK. Future financial incentives for PCU should be more specific for preventive care. Flexibility in when and how to spend incentives would maintain autonomy of people to act according to perceived need.

## 6 ETHICS APPROVAL

The study received ethical approval from the Human Subjects Ethics Sub‐Committee (HSESC) of the Hong Kong Polytechnic University (HSESC Reference Number: HSEARS20180629002).

## CONFLICT OF INTEREST

The authors declare they have no conflict of interest.

## AUTHOR CONTRIBUTIONS

QL developed the topic guide, conducted the focus‐group discussion, analysed the qualitative data and wrote the first draft of the manuscript. WL analysed the qualitative data and contributed to the draft of the results. SM contributed to the development of the topic guide, interpretation of the results and the draft of the manuscript. MY, RS and JL contributed to the critical discussion of the results and edition on the manuscript. JXL designed the qualitative study, developed the topic guide, conducted the focus‐group discussion, interpreted the results and drafted the manuscript.

## Data Availability

Research data are not shared due to ethical issues. ‘The data that support the findings of this study are available on request from the corresponding author. The data are not publicly available due to privacy or ethical restrictions’.

## APPENDIX 1

### TOPIC GUIDE USED IN THE FOCUS‐GROUP DISCUSSION

#### General Objectives

1. To explore elderly's views or experience of using financial incentives for preventive care;
2. To explore factors that affect use of financial incentives for preventive care among elderly.

#### Topic guides

##### Opinion and experience of using preventive care

1. First of all, can you share with us what is your understanding of preventive care?
2. What do you know about the types of preventive care for elderly?
3. What preventive care do you need? Why?
4. Do you use any preventive services? Where? How frequent? How do you pay for it?

[If the participants do not mention the following preventive care]

1. Have you had vaccination? Please tell us more about it.
2. Have you had a general health check? Please tell us more about it.
3. Have you had a comprehensive eye examination? Please tell us more about it.

[For those who DON’T have experience in using any of the above preventive care]

1. Could you please tell me why did you never use preventive services? (Note: cost may be one of the issues)
2. Could you share in what ways/what factors will encourage you to use these preventive services?
3. [Ask all participants] When you consider whether to use preventive service, is cost your concerning factor? How does it affect your decision?

[**Sum up**] Do you have any additional things you would like to share with us regarding your experience of using preventive care?

##### Opinions about financial incentives for preventive care

Currently, the government provides some financial incentive to encourage elderly to uptake more preventive care such as health check‐up, comprehensive eye examination and vaccination. For example, a health‐care voucher of HK$2000 per year is given to elderly aged 65 years or older, with accumulation limit of HK$5000. Another example is the provision of subsidized influenza vaccination to people aged 50 years or older. In 2017‐18, the government subsidy fee was HK$210 per dose. For the elderly, they can get the vaccination for free or with a very low price.

1. Does any of these financial subsidies mentioned above, such as flu vaccination subsidy scheme/ health‐care voucher, encourage you to use more preventive services? How does these encourage you?
2. What do you want the financial incentive to be? Or what do you like the financial incentive to be?

[type of financial incentives may include: A. To allocate a certain amount from the current health‐care voucher for preventive care; B. Inject additional amount to the current voucher but does not specify whether it is used for preventive care; C. Introduce designated vouchers specific for preventive care]

[At the beginning, keep the discussion as open as possible. As the interview proceeds, more specific questions regarding different characteristics of the financial incentives can be asked whether they are not prompted by participants’ response]

1. [If anyone mentions A]: How much do you think should set aside for preventive care? Why? Any different views? How does it affect your utilization of preventive care?
2. [If no one mentions A]: Some mention about setting a certain proportion on the current health‐care voucher just for preventive care, what is your view on this? How will it affect your utilization of preventive care?
3. [If anyone mentions B]: How much do you think the government should inject to the current health‐care voucher? Why? Any different views from the others? How does it affect your utilization of preventive care?
4. [If no one mentions B]: Some suggest the government should inject addition amount to the current health‐care voucher, what are your view on this? How will it affect your utilization of preventive care?
5. There are various types of financial incentive for preventive services, for example government direct subsidy, for example the flu vaccination; or it could be launching designated health‐care voucher for preventive services similar to the current health‐care voucher; or it could be in cash handout. What do you think of these different forms of financial incentive? (type)
6. Who do you think the government should provide these financial incentives for preventive care? (recipient) Why?
7. If the government was to provide financial incentives for preventive care, would this encourage you to uptake preventive care? (value)

If yes, what is the minimum subsidy amount that will make you consider using these preventive services? [If the elder cannot give a specific amount, please ask them the maximum price they are willing to co‐pay] in response to the following three services:

If No, what do you think should be the minimum subsidy amount to subsidize others to use these preventive services?

1. When do you think the financial incentives should be delivered to you? (schedule**)**
2. Some advocated that certain preventive service should introduce financial subsidy measures. Take health‐care voucher as an example, what do you think of designing specific health‐care vouchers for specific preventive service? (designated)
3. Some advocated putting a limit on the voucher amount, which elders could use for specific preventive care such as comprehensive eye examination. [here, moderator can mention that the voucher amount claimed by an optometrist is HK$2000 every two years] What are your opinions on this? (charge limit)
4. The current accumulation limit of the health‐care voucher is HK$5000. Suppose a designated health‐care voucher were to introduce for preventive services, what do you think should be the accumulative limit? (accumulative ceiling)
5. Do you think there should be an expiry date on the voucher for preventive care? For example, the health‐care vouchers may expire if you don't use it before a specific date. (effective date) What do you think are the advantages and disadvantages?
6. Regarding the current elderly health‐care voucher, if the current health‐care voucher was to improve to encourage the elders to use preventive care, what are your suggestions to improve the current design of it?

[**Sum up**] Regarding financial incentive for preventive care, do you have any additional ideas or opinions to share with us?
